# Crystal structure of 4-[(3-methyl­but-3-eno­yl)­oxy]phenyl 4-*n*-hexyl­oxybenzoate

**DOI:** 10.1107/S2056989017008568

**Published:** 2017-06-20

**Authors:** Lyudmila G. Kuz’mina, Ivan I. Konstantinov, Andrei V. Churakov, Mger A. Navasardyan

**Affiliations:** a Institute of General and Inorganic Chemistry RAS, 31 Leninskii prosp., Moscow 119991, Russian Federation; b Institute of Petrochemical Synthesis RAS, 29 Leninskii prosp., Moscow 119991, Russian Federation

**Keywords:** crystal structure, phenyl­benzoate, DSC study

## Abstract

The structure of the title compound, C_23_H_26_O_5_ or CH_2_=C(CH_3_)—C(O)O—C_6_H_4_—O(O)C—C_6_H_4_—OC_6_H_13_, has been determined. The mol­ecule is non-planar and the dihedral angle between the phenyl rings is 50.72 (4)°. The crystal packing differs from those typical for mesogenic compounds. Only a weak directional inter­action of the C—H⋯O type combines mol­ecules in endless chains running along the *a* axis.

## Abstract 2

The mol­ecule is non-planar and the dihedral angle between the phenyl rings is 50.72 (4)°. Only a weak directional inter­action of the C—H⋯O type combines mol­ecules in infinite chains running along the *a* axis.

## Chemical context   

Phenyl­benzoates bearing a rather long aliphatic substituent at the benzene ring are potentially mesogenic compounds. On melting, these compounds often form smectic or nematic phases. Cases where these compounds exhibit a monotropic mesomorphism, *i.e.* do not form the mesophase on melting but instead form it on cooling the isotropic melt, are also known. The structural studies of these compounds are of great inter­est as these investigations make it possible to clarify the structure of the mesophase and propose a mechanism of phase transitions in a crystal-mesophase-isotropic system.
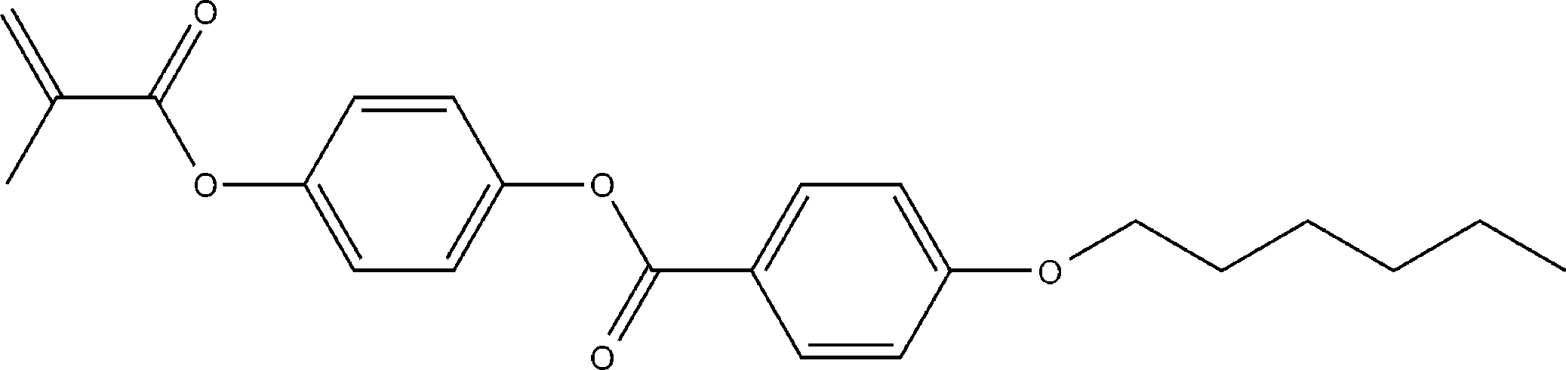


In this work we performed an X-ray structural determination and DSC study of the title compound. According to DSC the compound is non–mesomorphic, exhibiting three solid-state modifications: *Cr_III_* 367.7 K *Iso* 350.6 K *Cr_II_* 349.9 K *Cr_I_*.

## Structural commentary   

**Figure 1:**
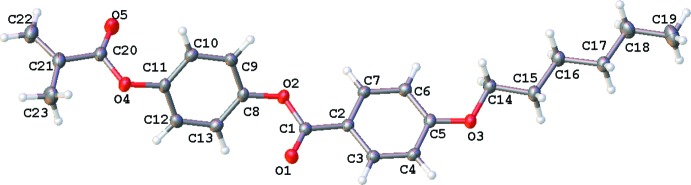
The mol­ecular structure of the title compound. Displacement ellipsoids are shown at the 50% probability level. The H atoms are presented as a small spheres of arbitrary radius.

The unit cell contains one independent mol­ecule whose structure is shown in Fig. 1 ▸. The mol­ecule is non-planar. Five planar fragments can be selected in it, *viz.* benzene rings C8–C13 (plane I) and C2–C7 (plane II), ester groups C2/C1/O1/O2 (plane III) and O4/O5/C20/C21 (plane IV) and the hex­yloxy group O3/C14–C19 (plane V). The dihedral angles between the planes I/II, II/III, II/V, I/III and I/IV are 50.72 (4), 4.84 (5), 7.05 (3), 52.82 (4) and 55.50 (5)°, respectively. According to the CSD Groom *et al.*, 2016 ▸), the dihedral angle between the planes of the benzene rings in phenyl­benzoates varies over a rather wide range (30–90°) having a normal distribution with the maximum at ∼60°. The obtained values of the dihedral angles in the structure provide evidence that the ester group C2/C1/O1/O2 is in a π-conjugation with the benzene ring C2–C7 bonded to the ester group through a C—C bond and is out of π-conjugation with the benzene ring C8–C13 bonded with it through a C—O bond. The same feature is characteristic of the second ester group bounded with the benzene ring C8–C13 through a C–O bond. This group is also strongly rotated from the plane of the indicated benzene ring and does not participate in conjugation with it. As is usual for liquid crystal compounds with a rather long alk­yloxy chain O—C_*n*_H_2*n*+1_ (*n* > 4), this substituent has an extended structure and its plane is nearly coplanar with the plane of the corresponding benzene ring.

## Supra­molecular features   

**Figure 2:**
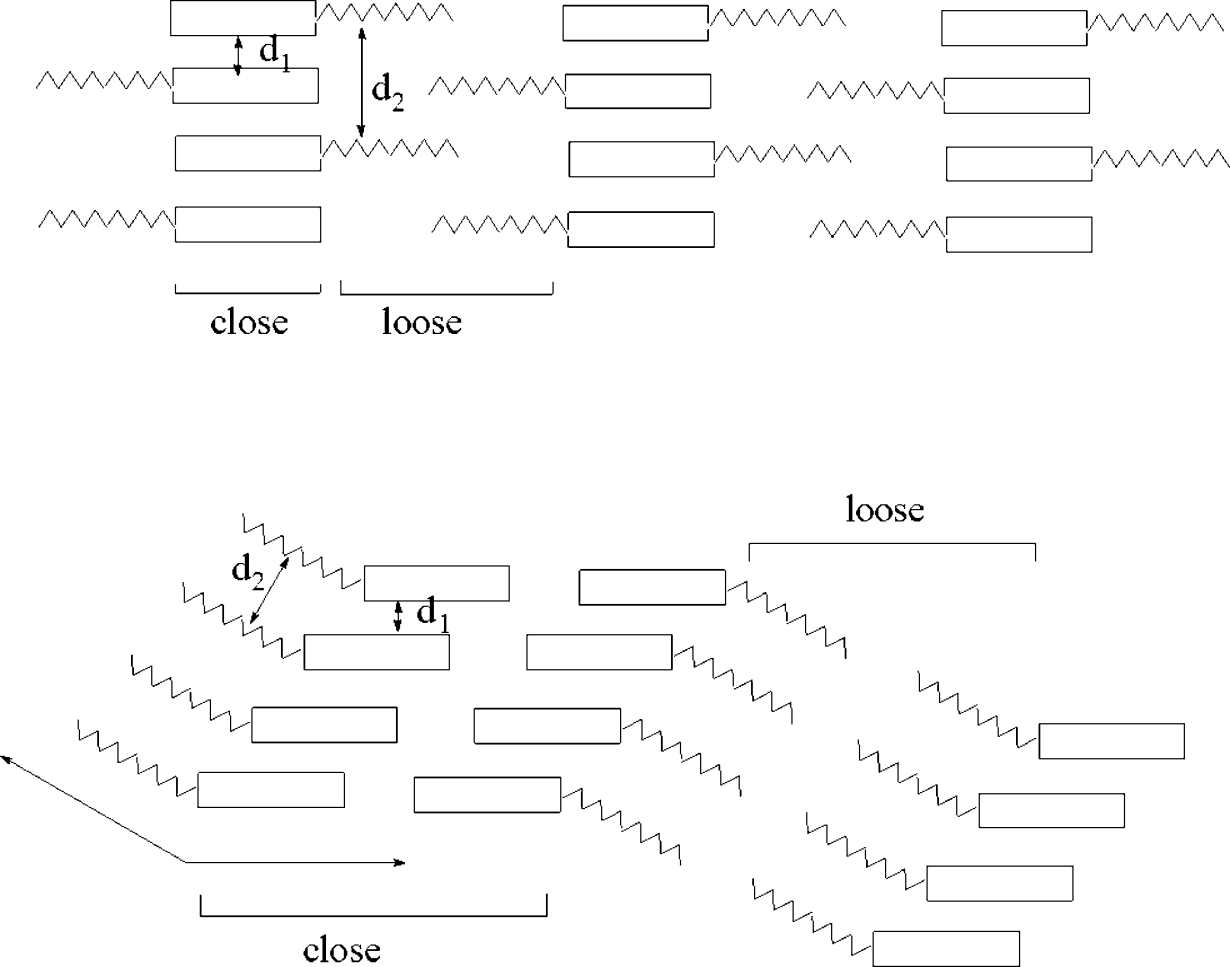
Two variants of crystal packing for mesogenic compounds; rectangles denote aromatic fragments and zigzags denote aliphatic side chains; *d*_2_ > *d*_1_.

It is known that crystal packing of mesogenic compounds is characterized by certain features, one of which is the separation of the packing into alternating aromatic and aliphatic areas, as shown in Fig. 2 ▸. Another feature is that the aromatic areas are closely packed, whereas the aliphatic areas have a very loose crystal packing. The close packing is formed as a result of many non-directional van der Waals and weak directional inter­actions. The most typical directional inter­actions are weak hydrogen bonds C—H⋯O/N, π–π stacking and C—H⋯π inter­actions (Nangia, 2002 ▸; Janiak, 2000 ▸; Chen *et al.*, 2009 ▸), as well as usual hydrogen bonds. The loose aliphatic areas involve only a few van der Waals contacts. These peculiarities bring about specific melting of the mesogenic compounds. Upon a rise in temperature, melting starts from the loose aliphatic areas, whereas the aromatic areas retain their ordering over a certain time, resulting in mesophase formation. All these peculiarities have been observed in the crystal packing of alkyl- and alkyl­oxycyano­biphenyls (Kuz’mina & Kucherepa, 2011 ▸; Kuz’mina *et al.*, 2012 ▸), alkyl­oxybenzoic acids (Kuz’mina *et al.*, 2009 ▸), *n*-(alkyl­oxybenzil­idene)-*n′*-tolyidines (Kuz’mina *et al.*, 2016 ▸) and phenyl­benzoates (Konstanti­nov *et al.*, 2013 ▸; Kuz’mina *et al.*, 2014 ▸), which represent a precursor of the mesophase.

**Figure 3:**
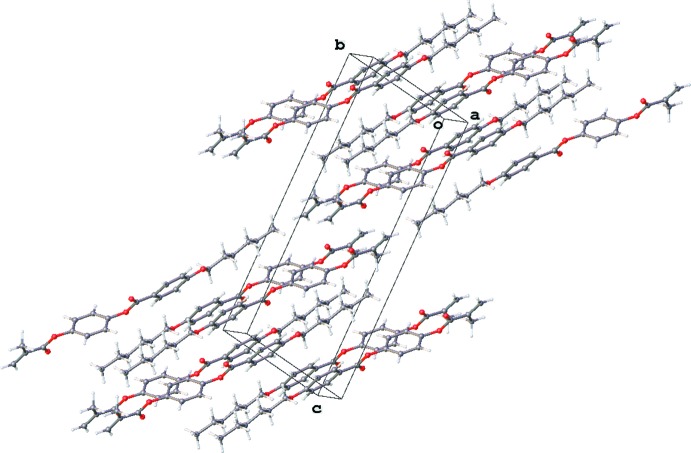
The crystal packing of the title compound.

The crystal packing of the title compound is shown in Fig. 3 ▸. Both aforementioned features of mesogenic compound crystal packing are lacking in the compound. An analysis of the inter­molecular distances of the aliphatic chain atoms indicates that there are no loosely packed areas, which explains lacking the mesomorphism for this compound.

**Figure 4:**
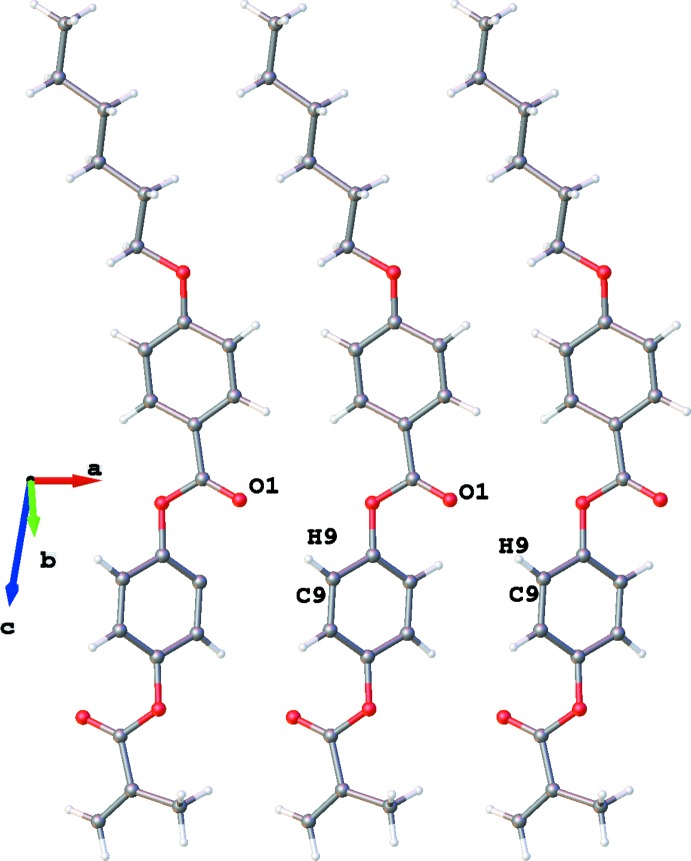
Translation related (along the *a* axis) mol­ecules.

In the crystal, only C9—H9⋯O1 contacts between translationally (along the *a* axis) related mol­ecules may be considered to be weak hydrogen bonds (Table 1 ▸, Fig. 4 ▸). The H9⋯O1 distances are equal to 2.47 Å, which corresponds to common values. The H9 atom is rather acidic to participate in a weak hydrogen bond since it is situated at the *ortho* position to the accepting ester group. A detailed analysis of the crystal packing did not reveal contacts that could be considered to be weak directional inter­actions of other types.

Inter­estingly, on cooling the isotropic melt of the compound, the formed crystal modifications *Cr_II_* and *Cr_I_* differ from that found in the crystal modification grown from solution at room temperature. Nevertheless, these modifications are also non-mesomorphous. The lack of mesomorphism of the compound in all crystal modifications may be explained by the occurrence of the branched metacryl group at the benzene ring C8–C13 that efficiently fills the adjacent areas in the crystal packing, thus restricting the displacement of the aliphatic chains.

## Synthesis and crystallization   

The compound was prepared by the reaction of 4-*n*-hexyl­oxybenzoic acid with 4-methacryloyloxyphenol using *N*,*N*-di­cyclo­hexyl­carbodi­imide in di­chloro­methane solution according to the procedure described by Hassner & Alexanian (1978 ▸). The product was purified by column chromatography and then recrystallized from acetone. Its purity was checked by thin-layer chromatography.

## Refinement   

Crystal data, data collection and structure refinement details are summarized in Table 2 ▸. All H atoms were located from a difference Fourier synthesis and refined isotropically without constrains and restrains.

## Supplementary Material

Crystal structure: contains datablock(s) I. DOI: 10.1107/S2056989017008568/rk2436sup1.cif

Structure factors: contains datablock(s) I. DOI: 10.1107/S2056989017008568/rk2436Isup2.hkl

Supporting information file. DOI: 10.1107/S2056989017008568/rk2436Isup3.cml

CCDC reference: 1554948

Additional supporting information:  crystallographic information; 3D view; checkCIF report

## Figures and Tables

**Table 1:** Hydrogen-bond geometry (Å, °)

*D*—H⋯*A*	*D*—H	H⋯*A*	*D*⋯*A*	*D*—H⋯*A*
C9—H9⋯O1^i^	0.943 (16)	2.471 (16)	3.3774 (15)	161.3 (12)

Symmetry code: (i) 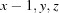.

**Table 2:** Experimental details

Crystal data	
Chemical formula	C_23_H_26_O_5_
*M*_r_	382.44
Crystal system, space group	Triclinic, *P* 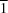
Temperature (K)	150
*a*, *b*, *c* (Å)	5.6805 (3), 8.3846 (5), 21.4864 (12)
α, β, γ (°)	99.191 (1), 92.719 (1), 91.701 (1)
*V* (Å^3^)	1008.37 (10)
*Z*	2
Radiation type	Mo *K*α
μ (mm^−1^)	0.09
Crystal size (mm)	0.48 × 0.14 × 0.08

Data collection	
Diffractometer	Bruker SMART APEXII CCD area detector
Absorption correction	Multi-scan (SADAB; Bruker, 2008 ▸)
*T*_min_, *T*_max_	0.660, 0.746
No. of measured, independent and observed [*I* > 2σ(*I*)] reflections	11249, 5330, 3849
*R*_int_	0.025
(sin θ/λ)_max_ (Å^−1^)	0.682

Refinement	
*R*[*F* ^2^ > 2σ(*F* ^2^)], *wR*(*F* ^2^), *S*	0.045, 0.117, 1.04
No. of reflections	5330
No. of parameters	357
H-atom treatment	All H-atom parameters refined
Δρ_max_, Δρ_min_ (e Å^−3^)	0.32, −0.23

Computer programs: *SMART* and *SAINT* (Bruker, 2009 ▸), *SHELXS97* and *SHELXL97* (Sheldrick, 2008 ▸) and OLEX2 (Dolomanov *et al.*, 2009 ▸).

## supplementary crystallographic information

### Crystal data

**Table:**

C_23_H_26_O_5_	*Z* = 2
*M**_r_* = 382.44	*F*(000) = 408
Triclinic, *P*1	*D*_x_ = 1.260 Mg m^−^^3^
*a* = 5.6805 (3) Å	Mo *K*α radiation, λ = 0.71073 Å
*b* = 8.3846 (5) Å	Cell parameters from 2709 reflections
*c* = 21.4864 (12) Å	θ = 2.5–30.3°
α = 99.191 (1)°	µ = 0.09 mm^−^^1^
β = 92.719 (1)°	*T* = 150 K
γ = 91.701 (1)°	Prism, colourless
*V* = 1008.37 (10) Å^3^	0.48 × 0.14 × 0.08 mm

### Data collection

**Table:**

Bruker SMART APEXII CCD area detector diffractometer	5330 independent reflections
Radiation source: fine-focus sealed tube	3849 reflections with *I* > 2σ(*I*)
Graphite monochromator	*R*_int_ = 0.025
φ– and ω–scans	θ_max_ = 29.0°, θ_min_ = 1.9°
Absorption correction: multi-scan (SADAB; Bruker, 2008)	*h* = −7→7
*T*_min_ = 0.660, *T*_max_ = 0.746	*k* = −11→11
11249 measured reflections	*l* = −28→29

### Refinement

**Table:**

Refinement on *F*^2^	Primary atom site location: structure-invariant direct methods
Least-squares matrix: full	Secondary atom site location: difference Fourier map
*R*[*F*^2^ > 2σ(*F*^2^)] = 0.045	Hydrogen site location: inferred from neighbouring sites
*wR*(*F*^2^) = 0.117	All H-atom parameters refined
*S* = 1.04	*w* = 1/[σ^2^(*F*_o_^2^) + (0.0585*P*)^2^ + 0.0431*P*] where *P* = (*F*_o_^2^ + 2*F*_c_^2^)/3
5330 reflections	(Δ/σ)_max_ < 0.001
357 parameters	Δρ_max_ = 0.32 e Å^−^^3^
0 restraints	Δρ_min_ = −0.23 e Å^−^^3^

### Special details

**Table:**

Geometry. All s.u.'s (except the s.u. in the dihedral angle between two l.s. planes) are estimated using the full covariance matrix. The cell s.u.'s are taken into account individually in the estimation of s.u.'s in distances, angles and torsion angles; correlations between s.u.'s in cell parameters are only used when they are defined by crystal symmetry. An approximate (isotropic) treatment of cell s.u.'s is used for estimating s.u.'s involving l.s. planes.
Refinement. Refinement of *F*^2^ against ALL reflections. The weighted *R*–factor w*R* and goodness of fit *S* are based on *F*^2^, conventional *R*–factors *R* are based on *F*, with *F* set to zero for negative *F*^2^. The threshold expression of *F*^2^ > 2σ(*F*^2^) is used only for calculating *R*–factors(gt) etc. and is not relevant to the choice of reflections for refinement. *R*–factors based on *F*^2^ are statistically about twice as large as those based on *F*, and *R*–factors based on ALL data will be even larger.

### Fractional atomic coordinates and isotropic or equivalent isotropic displacement parameters (Å^2^)

**Table:**

	*x*	*y*	*z*	*U*_iso_*/*U*_eq_	
O1	1.32606 (15)	0.12065 (11)	0.13553 (4)	0.0286 (2)	
O2	0.97617 (15)	0.04386 (10)	0.16947 (4)	0.0257 (2)	
O3	0.99053 (15)	−0.41394 (10)	−0.09472 (4)	0.0253 (2)	
O4	1.01986 (15)	0.53782 (11)	0.37446 (4)	0.0278 (2)	
O5	0.68226 (17)	0.46661 (13)	0.41579 (5)	0.0419 (3)	
C1	1.1518 (2)	0.03297 (14)	0.12803 (5)	0.0208 (2)	
C2	1.0995 (2)	−0.09530 (14)	0.07307 (5)	0.0197 (2)	
C3	1.2689 (2)	−0.11683 (15)	0.02774 (6)	0.0218 (3)	
H3	1.418 (3)	−0.0549 (17)	0.0345 (7)	0.034 (4)*	
C4	1.2282 (2)	−0.22457 (15)	−0.02734 (6)	0.0229 (3)	
H4	1.339 (3)	−0.2381 (17)	−0.0599 (7)	0.032 (4)*	
C5	1.0156 (2)	−0.31488 (14)	−0.03819 (5)	0.0207 (2)	
C6	0.8480 (2)	−0.29893 (14)	0.00758 (6)	0.0222 (3)	
H6	0.704 (2)	−0.3634 (17)	0.0006 (6)	0.026 (3)*	
C7	0.8906 (2)	−0.18837 (14)	0.06278 (6)	0.0214 (2)	
H7	0.775 (2)	−0.1768 (16)	0.0936 (6)	0.025 (3)*	
C8	0.9940 (2)	0.16953 (14)	0.22149 (5)	0.0218 (3)	
C9	0.8023 (2)	0.26700 (15)	0.22967 (6)	0.0239 (3)	
H9	0.674 (3)	0.2492 (16)	0.1996 (7)	0.031 (4)*	
C10	0.8037 (2)	0.38796 (15)	0.28172 (6)	0.0244 (3)	
H10	0.673 (2)	0.4579 (16)	0.2881 (6)	0.025 (3)*	
C11	0.9979 (2)	0.40909 (15)	0.32390 (6)	0.0232 (3)	
C12	1.1896 (2)	0.31167 (16)	0.31564 (6)	0.0249 (3)	
H12	1.315 (2)	0.3316 (16)	0.3464 (6)	0.028 (4)*	
C13	1.1875 (2)	0.18957 (15)	0.26387 (6)	0.0243 (3)	
H13	1.324 (2)	0.1234 (16)	0.2570 (6)	0.025 (3)*	
C14	0.7688 (2)	−0.50073 (15)	−0.11192 (6)	0.0237 (3)	
H14A	0.642 (2)	−0.4245 (17)	−0.1094 (6)	0.027 (4)*	
H14B	0.738 (2)	−0.5776 (16)	−0.0828 (6)	0.026 (3)*	
C15	0.7830 (2)	−0.58681 (16)	−0.17877 (6)	0.0261 (3)	
H15A	0.927 (3)	−0.6575 (17)	−0.1810 (6)	0.031 (4)*	
H15B	0.810 (3)	−0.5070 (18)	−0.2066 (7)	0.036 (4)*	
C16	0.5590 (2)	−0.68682 (16)	−0.20221 (6)	0.0261 (3)	
H16A	0.422 (3)	−0.6186 (18)	−0.1961 (7)	0.032 (4)*	
H16B	0.532 (3)	−0.7710 (18)	−0.1752 (7)	0.036 (4)*	
C17	0.5631 (2)	−0.76731 (16)	−0.27084 (6)	0.0262 (3)	
H17A	0.583 (2)	−0.6819 (18)	−0.2984 (7)	0.033 (4)*	
H17B	0.702 (3)	−0.8369 (18)	−0.2756 (7)	0.038 (4)*	
C18	0.3397 (3)	−0.86774 (19)	−0.29400 (7)	0.0355 (3)	
H18A	0.310 (3)	−0.947 (2)	−0.2637 (8)	0.050 (5)*	
H18B	0.204 (3)	−0.798 (2)	−0.2900 (8)	0.049 (5)*	
C19	0.3486 (4)	−0.9629 (2)	−0.35978 (8)	0.0484 (4)	
H19A	0.374 (3)	−0.893 (2)	−0.3915 (9)	0.060 (5)*	
H19B	0.200 (4)	−1.030 (2)	−0.3717 (9)	0.069 (6)*	
H19C	0.480 (4)	−1.039 (2)	−0.3619 (9)	0.066 (6)*	
C20	0.8557 (2)	0.55349 (16)	0.41884 (6)	0.0263 (3)	
C21	0.9248 (2)	0.69069 (16)	0.47011 (6)	0.0287 (3)	
C22	0.7842 (3)	0.7171 (2)	0.51894 (7)	0.0378 (3)	
H22A	0.827 (3)	0.804 (2)	0.5527 (8)	0.048 (5)*	
H22B	0.652 (3)	0.648 (2)	0.5221 (8)	0.050 (5)*	
C23	1.1432 (3)	0.7870 (2)	0.46524 (8)	0.0422 (4)	
H23A	1.282 (3)	0.719 (2)	0.4610 (9)	0.064 (6)*	
H23B	1.173 (3)	0.875 (2)	0.5006 (9)	0.060 (5)*	
H23C	1.132 (3)	0.837 (2)	0.4253 (9)	0.058 (5)*	

### Atomic displacement parameters (Å^2^)

**Table:**

	*U*^11^	*U*^22^	*U*^33^	*U*^12^	*U*^13^	*U*^23^
O1	0.0254 (5)	0.0325 (5)	0.0256 (5)	−0.0076 (4)	0.0021 (4)	−0.0017 (4)
O2	0.0248 (4)	0.0263 (5)	0.0232 (4)	−0.0036 (4)	0.0055 (3)	−0.0048 (4)
O3	0.0258 (4)	0.0263 (5)	0.0216 (4)	−0.0015 (4)	0.0024 (3)	−0.0028 (4)
O4	0.0283 (5)	0.0291 (5)	0.0231 (4)	−0.0031 (4)	0.0051 (4)	−0.0053 (4)
O5	0.0341 (6)	0.0567 (7)	0.0302 (5)	−0.0122 (5)	0.0084 (4)	−0.0072 (5)
C1	0.0213 (6)	0.0217 (6)	0.0199 (6)	0.0020 (5)	0.0010 (4)	0.0044 (5)
C2	0.0208 (6)	0.0191 (6)	0.0194 (6)	0.0031 (4)	0.0015 (4)	0.0029 (4)
C3	0.0204 (6)	0.0226 (6)	0.0224 (6)	−0.0001 (5)	0.0012 (5)	0.0036 (5)
C4	0.0221 (6)	0.0249 (6)	0.0222 (6)	0.0031 (5)	0.0055 (5)	0.0034 (5)
C5	0.0246 (6)	0.0177 (6)	0.0196 (6)	0.0035 (5)	0.0006 (5)	0.0019 (4)
C6	0.0211 (6)	0.0210 (6)	0.0242 (6)	−0.0008 (5)	0.0020 (5)	0.0028 (5)
C7	0.0192 (6)	0.0238 (6)	0.0210 (6)	0.0016 (5)	0.0032 (5)	0.0023 (5)
C8	0.0246 (6)	0.0203 (6)	0.0191 (6)	−0.0036 (5)	0.0042 (5)	−0.0006 (5)
C9	0.0209 (6)	0.0287 (6)	0.0213 (6)	−0.0023 (5)	0.0002 (5)	0.0020 (5)
C10	0.0229 (6)	0.0262 (6)	0.0237 (6)	0.0028 (5)	0.0039 (5)	0.0020 (5)
C11	0.0255 (6)	0.0235 (6)	0.0189 (6)	−0.0035 (5)	0.0046 (5)	−0.0013 (5)
C12	0.0232 (6)	0.0305 (7)	0.0203 (6)	−0.0019 (5)	−0.0011 (5)	0.0034 (5)
C13	0.0240 (6)	0.0250 (6)	0.0241 (6)	0.0030 (5)	0.0028 (5)	0.0037 (5)
C14	0.0248 (6)	0.0227 (6)	0.0227 (6)	−0.0004 (5)	0.0018 (5)	0.0010 (5)
C15	0.0299 (7)	0.0254 (6)	0.0219 (6)	−0.0029 (5)	0.0018 (5)	0.0012 (5)
C16	0.0286 (7)	0.0250 (6)	0.0239 (6)	−0.0029 (5)	0.0021 (5)	0.0017 (5)
C17	0.0298 (7)	0.0232 (6)	0.0248 (6)	−0.0006 (5)	0.0016 (5)	0.0013 (5)
C18	0.0364 (8)	0.0336 (8)	0.0337 (8)	−0.0090 (6)	−0.0022 (6)	−0.0001 (6)
C19	0.0585 (11)	0.0436 (10)	0.0374 (9)	−0.0066 (9)	−0.0135 (8)	−0.0043 (8)
C20	0.0259 (6)	0.0315 (7)	0.0205 (6)	0.0037 (5)	0.0013 (5)	0.0005 (5)
C21	0.0317 (7)	0.0306 (7)	0.0225 (6)	0.0061 (5)	−0.0016 (5)	−0.0001 (5)
C22	0.0430 (9)	0.0423 (9)	0.0262 (7)	0.0053 (7)	0.0040 (6)	−0.0012 (6)
C23	0.0417 (9)	0.0399 (9)	0.0389 (9)	−0.0093 (7)	0.0046 (7)	−0.0113 (7)

### Geometric parameters (Å, º)

**Table:**

O1—C1	1.2044 (13)	C13—H13	0.968 (14)
O2—C1	1.3636 (14)	C14—H14A	0.976 (14)
O2—C8	1.4059 (13)	C14—H14B	0.986 (14)
O3—C5	1.3563 (14)	C14—C15	1.5080 (17)
O3—C14	1.4382 (15)	C15—H15A	1.022 (15)
O4—C11	1.4019 (14)	C15—H15B	0.980 (16)
O4—C20	1.3594 (15)	C15—C16	1.5221 (17)
O5—C20	1.2007 (15)	C16—H16A	0.983 (15)
C1—C2	1.4770 (16)	C16—H16B	0.996 (16)
C2—C3	1.3965 (16)	C16—C17	1.5221 (18)
C2—C7	1.3897 (16)	C17—H17A	1.008 (15)
C3—H3	0.971 (15)	C17—H17B	0.994 (16)
C3—C4	1.3749 (17)	C17—C18	1.5199 (19)
C4—H4	0.959 (15)	C18—H18A	1.018 (18)
C4—C5	1.3960 (16)	C18—H18B	0.982 (18)
C5—C6	1.3950 (16)	C18—C19	1.512 (2)
C6—H6	0.959 (14)	C19—H19A	0.98 (2)
C6—C7	1.3903 (16)	C19—H19B	1.00 (2)
C7—H7	0.951 (14)	C19—H19C	0.99 (2)
C8—C9	1.3822 (17)	C20—C21	1.4894 (18)
C8—C13	1.3823 (17)	C21—C22	1.3427 (19)
C9—H9	0.944 (14)	C21—C23	1.477 (2)
C9—C10	1.3841 (17)	C22—H22A	0.961 (17)
C10—H10	0.961 (14)	C22—H22B	0.945 (18)
C10—C11	1.3828 (17)	C23—H23A	0.99 (2)
C11—C12	1.3819 (18)	C23—H23B	0.977 (18)
C12—H12	0.942 (14)	C23—H23C	1.013 (19)
C12—C13	1.3856 (17)		
			
C1—O2—C8	118.12 (9)	C15—C14—H14B	111.3 (8)
C5—O3—C14	118.59 (9)	C14—C15—H15A	108.8 (8)
C20—O4—C11	119.76 (10)	C14—C15—H15B	109.3 (9)
O1—C1—O2	123.19 (11)	C14—C15—C16	112.14 (11)
O1—C1—C2	124.69 (11)	H15A—C15—H15B	106.4 (12)
O2—C1—C2	112.11 (10)	C16—C15—H15A	111.0 (8)
C3—C2—C1	116.71 (10)	C16—C15—H15B	109.1 (9)
C7—C2—C1	124.17 (10)	C15—C16—H16A	109.6 (8)
C7—C2—C3	119.05 (11)	C15—C16—H16B	110.0 (8)
C2—C3—H3	120.4 (9)	H16A—C16—H16B	103.9 (12)
C4—C3—C2	120.91 (11)	C17—C16—C15	113.23 (11)
C4—C3—H3	118.7 (9)	C17—C16—H16A	110.4 (8)
C3—C4—H4	122.3 (8)	C17—C16—H16B	109.3 (8)
C3—C4—C5	119.83 (11)	C16—C17—H17A	109.4 (8)
C5—C4—H4	117.8 (8)	C16—C17—H17B	108.7 (9)
O3—C5—C4	115.06 (10)	H17A—C17—H17B	107.6 (12)
O3—C5—C6	124.98 (11)	C18—C17—C16	113.10 (11)
C6—C5—C4	119.96 (11)	C18—C17—H17A	108.3 (8)
C5—C6—H6	119.9 (8)	C18—C17—H17B	109.7 (8)
C7—C6—C5	119.54 (11)	C17—C18—H18A	108.5 (9)
C7—C6—H6	120.6 (8)	C17—C18—H18B	109.3 (10)
C2—C7—C6	120.65 (11)	H18A—C18—H18B	103.9 (14)
C2—C7—H7	120.0 (8)	C19—C18—C17	114.27 (14)
C6—C7—H7	119.3 (8)	C19—C18—H18A	108.3 (9)
C9—C8—O2	116.27 (10)	C19—C18—H18B	112.0 (10)
C9—C8—C13	121.80 (11)	C18—C19—H19A	112.4 (11)
C13—C8—O2	121.86 (11)	C18—C19—H19B	110.7 (11)
C8—C9—H9	119.1 (8)	C18—C19—H19C	110.7 (11)
C8—C9—C10	119.18 (11)	H19A—C19—H19B	109.3 (16)
C10—C9—H9	121.7 (8)	H19A—C19—H19C	107.2 (16)
C9—C10—H10	120.5 (8)	H19B—C19—H19C	106.2 (15)
C11—C10—C9	119.12 (11)	O4—C20—C21	110.19 (11)
C11—C10—H10	120.3 (8)	O5—C20—O4	123.39 (11)
C10—C11—O4	122.03 (11)	O5—C20—C21	126.41 (12)
C12—C11—O4	116.12 (11)	C22—C21—C20	117.14 (14)
C12—C11—C10	121.66 (11)	C22—C21—C23	123.96 (14)
C11—C12—H12	117.4 (8)	C23—C21—C20	118.89 (12)
C11—C12—C13	119.28 (12)	C21—C22—H22A	118.3 (10)
C13—C12—H12	123.3 (8)	C21—C22—H22B	121.8 (10)
C8—C13—C12	118.97 (12)	H22A—C22—H22B	119.7 (14)
C8—C13—H13	121.3 (8)	C21—C23—H23A	111.5 (11)
C12—C13—H13	119.6 (8)	C21—C23—H23B	113.1 (11)
O3—C14—H14A	109.4 (8)	C21—C23—H23C	109.5 (10)
O3—C14—H14B	110.7 (8)	H23A—C23—H23B	109.7 (15)
O3—C14—C15	107.03 (10)	H23A—C23—H23C	105.3 (15)
H14A—C14—H14B	108.0 (11)	H23B—C23—H23C	107.4 (15)
C15—C14—H14A	110.3 (8)		
			
O1—C1—C2—C3	1.43 (18)	C5—O3—C14—C15	174.48 (10)
O1—C1—C2—C7	−175.41 (12)	C5—C6—C7—C2	−0.80 (18)
O2—C1—C2—C3	−179.84 (10)	C7—C2—C3—C4	2.05 (17)
O2—C1—C2—C7	3.33 (16)	C8—O2—C1—O1	3.74 (17)
O2—C8—C9—C10	176.84 (10)	C8—O2—C1—C2	−175.02 (10)
O2—C8—C13—C12	−177.26 (10)	C8—C9—C10—C11	0.58 (18)
O3—C5—C6—C7	−177.51 (11)	C9—C8—C13—C12	−0.55 (18)
O3—C14—C15—C16	178.76 (10)	C9—C10—C11—O4	174.30 (11)
O4—C11—C12—C13	−175.19 (11)	C9—C10—C11—C12	−0.53 (19)
O4—C20—C21—C22	−177.29 (12)	C10—C11—C12—C13	−0.07 (19)
O4—C20—C21—C23	1.57 (17)	C11—O4—C20—O5	−3.94 (19)
O5—C20—C21—C22	2.4 (2)	C11—O4—C20—C21	175.79 (10)
O5—C20—C21—C23	−178.72 (15)	C11—C12—C13—C8	0.60 (18)
C1—O2—C8—C9	126.52 (12)	C13—C8—C9—C10	−0.05 (18)
C1—O2—C8—C13	−56.60 (15)	C14—O3—C5—C4	−174.72 (10)
C1—C2—C3—C4	−174.95 (11)	C14—O3—C5—C6	5.20 (17)
C1—C2—C7—C6	175.36 (11)	C14—C15—C16—C17	176.70 (11)
C2—C3—C4—C5	−0.46 (18)	C15—C16—C17—C18	179.72 (12)
C3—C2—C7—C6	−1.40 (17)	C16—C17—C18—C19	−174.03 (14)
C3—C4—C5—O3	178.14 (10)	C20—O4—C11—C10	60.01 (16)
C3—C4—C5—C6	−1.78 (18)	C20—O4—C11—C12	−124.89 (12)
C4—C5—C6—C7	2.40 (17)		

### Hydrogen-bond geometry (Å, º)

**Table:**

*D*—H···*A*	*D*—H	H···*A*	*D*···*A*	*D*—H···*A*
C9—H9···O1^i^	0.943 (16)	2.471 (16)	3.3774 (15)	161.3 (12)

Symmetry code: (i) *x*−1, *y*, *z*.
